# A Transverse Hamiltonian Approach to Infinitesimal Perturbation Analysis of Quantum Stochastic Systems

**DOI:** 10.3390/e25081179

**Published:** 2023-08-08

**Authors:** Igor G. Vladimirov

**Affiliations:** School of Engineering, Australian National University, Canberra, ACT 2601, Australia; igor.g.vladimirov@gmail.com

**Keywords:** quantum stochastic system, infinitesimal perturbation analysis, transverse Hamiltonian, integro-differential equation, 81Q93, 93E20, 81S25, 49J40, 47J20, 49K30, 37K05

## Abstract

This paper is concerned with variational methods for open quantum systems with Markovian dynamics governed by Hudson–Parthasarathy quantum stochastic differential equations. These QSDEs are driven by quantum Wiener processes of the external bosonic fields and are specified by the system Hamiltonian and system–field coupling operators. We consider the system response to perturbations in these operators and introduce a transverse Hamiltonian which encodes the propagation of the perturbations through the unitary system–field evolution. This approach provides an infinitesimal perturbation analysis tool which can be used for the development of optimality conditions in quantum control and filtering problems. As performance criteria, such settings employ quadratic (or more complicated) cost functionals of the system and field variables to be minimized over the energy and coupling parameters of system interconnections. We demonstrate an application of the transverse Hamiltonian variational approach to a mean square optimal coherent quantum filtering problem for a measurement-free field-mediated cascade connection of a quantum system with a quantum observer.

## 1. Introduction

In comparison with classical mechanics which considers macroscopic objects both in deterministic and stochastic settings, quantum mechanics is concerned with physical phenomena at atomic and subatomic scales and inherently incorporates randomness. In particular, the squared absolute value of the wave function of a quantum mechanical particle is interpreted as a probability density function, mixed quantum states are built of pure ones using randomization, and the latter is present in the model of quantum measurement [1,2]. However, in contrast to scalar-valued classical probability measures [3], quantum probability describes statistical properties of quantum variables by using quantum states in the form of density operators [4,5] on the same Hilbert space where those variables act as linear operators.

The noncommutativity and canonical commutation structures originating from the operator-valued nature of quantum variables and quantum states give rise to specific features of quantum probability such as the absence of a classical joint probability distribution and conditional expectations for a set of noncommuting quantum variables (whereas an individual self-adjoint operator has a well-defined marginal distribution). Furthermore, because the microscopic realm is less amenable to manipulation by conventional macroscopic tools (unlike, for example, coin tossing as a manageable “random number generator” for thought and practical experiments in classical probability theory), its natural time evolution makes the statistical properties of quantum systems particularly tied to their dynamics. While an isolated quantum system undergoes a reversible evolution according to a one-parameter unitary group generated by the system Hamiltonian, a more realistic open dynamics scenario [6] involves interaction of the system with its environment, which can include other quantum or classical systems, measuring devices, and external quantum fields such as quantized electromagnetic radiation.

The interaction of an open quantum system with its surroundings is accompanied by energy exchange between the subsystems, and gives rise to dissipative effects. This provides a way to influence statistical and dynamic properties of such systems by arranging them into quantum networks [7] and varying the energy and coupling parameters of the resulting interconnections, which can consist of a quantum plant coupled in a measurement-based or coherent (that is, measurement-free) fashion to a controller or observer. This paradigm is used in quantum control [8,9,10], which develops systematic methods for achieving stability, robustness with respect to unmodelled dynamics, and optimality in the sense of relevant performance criteria for quantum systems and their applications such as quantum optics [11,12] and quantum information processing [13].

An important part in these developments belongs to quantum filtering, which, similarly to its classical predecessor (see for example [14,15]), is concerned with mean square optimal real-time estimation of the internal variables of a quantum plant using a measurement-based quantum Kalman filter [16,17,18] or a qualitatively different coherent quantum observer [19,20]. The latter does not process classical observations and, in contrast to the classical filters, does not compute the conditional expectations of the plant variables (this conditioning and related Bayesian approaches are not applicable in the noncommutative quantum case, as mentioned above). Instead, the coherent quantum filter is driven by the output quantum fields of the plant to produce a quantum process, and its performance as an estimator of the plant variables can be optimized in the sense of minimizing the mean square value of the “estimation error” by varying the parameters of the Hamiltonian and coupling operators of the filter.

The optimization problems arising in coherent quantum filtering and control (with the latter considering more complicated feedback interconnections of a quantum plant and a quantum controller) involve physical realizability (PR) constraints [21,22] which originate from the canonical commutation structures of quantum dynamic variables, the energetics of open quantum systems, and unitarity in the augmented system–environment evolution. These features of open quantum dynamics are incorporated in Hudson–Parthasarathy quantum stochastic calculus [23,24] (see also [25]), which is often employed as a unified modelling framework in quantum filtering and control. In this approach, the external fields at the input of a quantum system of interest are represented by a multichannel quantum Wiener process with noncommuting components, which act on a symmetric Fock space and drive the quantum stochastic differential equation (QSDE) governing the system dynamics. In contrast to the classical SDEs with a standard Wiener process [26], the QSDE reflects the unitarity of the augmented system–field evolution, and its drift and dispersion terms (as well as the generator of Markovian quantum dynamics) are specified by the Hamiltonian and coupling operators. These operators, together with a scattering matrix which represents the photon exchange between the fields [24], describe the energetics of the quantum system and its interaction with the environment, and are usually functions (for example, polynomials or Weyl quantization integrals [27]) of the system variables. A particular form of this dependence and the commutation structure of the system variables affect the tractability of the quantum system under consideration.

In particular, quadratic dependence of the system Hamiltonian and linear dependence of the system–field coupling operators on the quantum mechanical position–momentum variables [28] lead to linear QSDEs for open quantum harmonic oscillators (OQHOs) [11,18], which play the role of building blocks in linear quantum control theory [10,21,29,30,31,32]. The dynamics of such systems are relatively well understood and are similar to the classical linear SDEs in a number of respects, including the preservation of the Gaussian nature of quantum states [33,34] in the case of vacuum input fields. However, the coherent quantum analogue [35] of the classical linear-quadratic Gaussian (LQG) control problem [36,37] for OQHOs is complicated by the above mentioned PR constraints on the state-space matrices of the quantum controller and by the impossibility of taking advantage of the classical estimation–actuation separation principle and classical conditional expectations with their variational properties [15].

One of the existing approaches to the coherent quantum LQG (CQLQG) control and coherent quantum filtering (CQF) problems employs the Frechet derivatives of the mean square cost (with respect to the state-space matrices subject to the PR constraints) in obtaining the optimality conditions [20,38] and for numerical optimization [39]. This approach takes into account the quantum nature of the underlying problem only through the PR constraints, being “classical” in all other respects, which has advantages from the viewpoint of relying on well-developed conventional optimization methods. However, a disadvantage of this approach is that it is limited to certain parametric classes of linear controllers and observers. In particular, the resulting optimality conditions do not provide information on whether nonlinear quantum controllers or observers can outperform linear ones for linear quantum plants. For this reason, the coherent quantum control and filtering problems require novel variational methods for their solution, which would be able to operate with sensitivity of the system dynamics and relevant cost functionals to perturbations over wider classes of the Hamiltonian and coupling operators in a “coordinate-free” fashion.

To this end, the present paper (several of its results were briefly announced in [40]) outlines a fully quantum variational method which allows the sensitivity of the internal and output variables of a nonlinear quantum stochastic system to be investigated with respect to arbitrary (that is, not only linear-quadratic) perturbations of the Hamiltonian and coupling operators. This approach is based on using a *transverse Hamiltonian*, defined as an auxiliary time-varying self-adjoint operator which encodes the propagation of such perturbations through the unitary system–field evolution. More precisely, the perturbation of the quantum stochastic flow, which describes the time evolution of a system operator (a function of the system variables), is expressed in terms of the commutator with the transverse Hamiltonian. The resulting *derivative processes* for system operators lead to an infinitesimal perturbation formula for quantum averaged performance criteria (such as the mean square cost functional) which is applicable to the development of optimality conditions in coherent quantum control and filtering problems over larger classes of controllers and observers. In particular, this approach allows the sensitivity of OQHOs with quadratic performance criteria to be studied with respect to general perturbations of the Hamiltonian and coupling operators. We demonstrate its application to a CQF problem for a field-mediated cascade connection of a quantum plant with a quantum observer. In fact, the transverse Hamiltonian method has already been employed in [41] to establish the local sufficiency of linear observers in the mean square optimal CQF problem [20] for linear quantum systems with respect to varying the Hamiltonian and coupling operators of the observer along linear combinations of the Weyl operators [27]. As an extended version of the conference paper [40], the present work provides its results along with detailed proofs. Furthermore, as part of the additional material we discuss the joint commutation structure, including the cross-commutation relations, of the unperturbed system variables and their perturbations (see Section 3), and elaborate on the case where the perturbations of the Hamiltonian and coupling operators are in the Weyl quantization form (see Section 6). Note that our approach is different from [42], which develops a quantum Hamilton–Jacobi–Bellman principle for the density operator instead of the dynamic variables in a measurement-based quantum feedback control problem. We also note a parallel between the perturbation analysis discussed in the present paper and the fluctuation–dissipation theorem [43].

The rest of this paper is organized as follows. Section 2 specifies the class of quantum stochastic systems under consideration. Section 3 discusses the sensitivity of the internal and output variables of OQHOs to parametric perturbations within the families of quadratic system Hamiltonians and linear system–field coupling operators. Section 4 returns to general QSDEs and introduces the transverse Hamiltonian associated with arbitrary perturbations of the Hamiltonian and coupling operators of the system. The transverse Hamiltonian is used in Section 5 for infinitesimal perturbation analysis of system operators. Section 6 extends the transverse Hamiltonian method to quantum averaged performance criteria. Section 7 applies this approach to a mean square optimal CQF problem. Section 8 makes concluding remarks.

## 2. Quantum Stochastic Systems Being Considered

We consider an open quantum system which interacts with an external multichannel bosonic field and is equipped with dynamic variables $X_{1}\left(t\right),\ldots ,X_{n}\left(t\right)$ evolving in time t⩾0 and assembled into a vector $X\left(t\right):={\left(X_{k}\left(t\right)\right)}_{1⩽k⩽n}$ (vectors are organized as columns unless indicated otherwise). These system variables are assumed to be self-adjoint operators on a composite system–field Hilbert space H⊗F, where ⊗ is the tensor product of spaces or operators (including the Kronecker product of matrices). Here, H is the initial space of the system, which provides a domain for $X_{1}\left(0\right),\ldots ,X_{n}\left(0\right)$, and F is a symmetric Fock space [24] for the action of an even number *m* of quantum Wiener processes $W_{1}\left(t\right),\ldots ,W_{m}\left(t\right)$. The latter are time-varying self-adjoint operators, which model the external fields and are assembled into a vector $W\left(t\right):={\left(W_{k}\left(t\right)\right)}_{1⩽k⩽m}$. Unlike the classical Brownian motion [26] in $R^{m}$, the quantum Wiener process *W* consists of noncommuting operator-valued components, and has a complex positive semi-definite Hermitian Ito matrix $\Omega :={\left(\omega_{jk}\right)}_{1⩽j,k⩽m}$ (identified with its tensor product $\Omega ⊗I_{F}$ with the identity operator $I_{F}$ on the Fock space F) for its future-pointing Ito increments dW:

(1) $$dW\left(t\right)dW{\left(t\right)}^{T}=\Omega dt,\;\Omega :=I_{m}+iJ.$$

Here, the transpose ${\left(\cdot \right)}^{T}$ acts on vectors and matrices of operators as if the latter were scalars, $I_{m}$ denotes the identity matrix of order *m*, and $i:=\sqrt{-1}$ is the imaginary unit. Furthermore, *J* is a real antisymmetric matrix of order *m* (we denote the subspace of such matrices by $A_{m}$) which specifies the canonical commutation relations (CCRs) for the constituent field processes $W_{1},\ldots ,W_{m}$:

(2) $$\left[dW\left(t\right),dW{\left(t\right)}^{T}\right]=2iJdt,\;J:=I_{m/2}⊗J,\;J:=\left[\begin{array}{cc} 0 & 1\\ -1 & 0 \end{array}\right]$$

with J spanning the one-dimensional subspace $A_{2}$ of antisymmetric (2×2)-matrices, which is an incremental form of the two-point CCRs

(3) $$[W\left(s\right),W{\left(t\right)}^{T}]=2i\min\left(s,t\right)J,\;s,t⩾0,$$

where the commutator [α,β]:=αβ−βα of linear operators α, β is extended to the commutator matrix $\left[\xi ,\eta^{T}\right]:={\left(\left[\xi_{j},\eta_{k}\right]\right)}_{1⩽j⩽r,1⩽k⩽s}=\xi \eta^{T}-{\left(\eta \xi^{T}\right)}^{T}$ for vectors $\xi :={\left(\xi_{j}\right)}_{1⩽j⩽r}$, $\eta :={\left(\eta_{k}\right)}_{1⩽k⩽s}$ of operators $\xi_{1},\ldots ,\xi_{r}$, $\eta_{1},\ldots ,\eta_{s}$. These CCRs are closely related to the continuous tensor–product structure of the Fock space [44] and are complemented by the commutativity between the Ito increments of *W* and adapted processes $\zeta :={\left(\zeta \left(t\right)\right)}_{t⩾0}$ taken at the same (or an earlier) moment of time:

(4) [ζ(s),dW(t)]=0,t⩾s⩾0.

The adaptedness of quantum processes on the system–field space H⊗F is understood with respect to a filtration ${\left(H_{t}\right)}_{t⩾0}$, where

(5) $$H_{t}:=H⊗F_{t},$$

and ${\left(F_{t}\right)}_{t⩾0}$ is the Fock space filtration, such that for any t⩾0 the operators $W_{j}\left(t\right)$ act effectively on $F_{t}$, while $X_{k}\left(t\right)$ act on the subspace $H_{t}$.

The energetics of the quantum system and its interaction with the external fields is specified by a system Hamiltonian H(t) and system–field coupling operators $L_{1}\left(t\right),\ldots ,L_{m}\left(t\right)$ which are time-varying self-adjoint operators organized as deterministic functions (for example, polynomials with constant coefficients) of the system variables $X_{1}\left(t\right),\ldots ,X_{n}\left(t\right)$ and assembled into a vector $L\left(t\right):={\left(L_{k}\left(t\right)\right)}_{1⩽k⩽m}$. Accordingly, the operators H(0), $L_{1}\left(0\right),\ldots ,L_{m}\left(0\right)$ act on the initial space H. Depending on the context, a system operator σ (a function of the initial system variables $X_{1}\left(0\right),\ldots ,X_{n}\left(0\right)$) on the initial space H will be identified with its extension $\sigma ⊗I_{F}$ to the system–field space H⊗F.

The system and the fields form a composite quantum system which evolves according to a quantum stochastic flow as described below. This evolution is specified at any time t⩾0 by a unitary operator U(t) on H⊗F governed by the following stochastic Schrödinger equation [23,24]:

(6) $$dU\left(t\right)=-\left(i\left(H_{0}dt+L_{0}^{T}dW\left(t\right)\right)+\frac{1}{2}L_{0}^{T}\Omega L_{0}dt\right)U\left(t\right),$$

with initial condition $U_{0}:=I_{H⊗F}$, and so U(t) captures the internal dynamics of the system and the system–field interaction over the time interval [0,t]. Here and in what follows, the subscript 0 marks the initial values of time-varying operators (as well as vectors or matrices of operators): $H_{0}:=H\left(0\right)$, $L_{0}:=L\left(0\right)$, $U_{0}:=U\left(0\right)$, while the time arguments are often omitted for the sake of brevity. In addition, the units are chosen such that the reduced Planck constant is ℏ=1. The quantum stochastic differential equation (QSDE) (6) corresponds to a particular yet important case of the identity scattering matrix in which there is no photon exchange between the fields and the gauge processes [24] can be eliminated from consideration. The term $L_{0}^{T}dW$ in (6) can be interpreted as an incremental Hamiltonian of the system–field interaction, while $\frac{1}{2}L_{0}^{T}\Omega L_{0}dt$ involves the quantum Ito matrix Ω from (1) and counterbalances the Ito correction term $dUdU^{†}=L_{0}^{T}\Omega L_{0}dt$ (with ${\left(\cdot \right)}^{†}$ denoting the operator adjoint) in the differential relation $d\left(UU^{†}\right)=\left(dU\right)U^{†}+UdU^{†}+dUdU^{†}=0$ that describes the preservation of the co-isometry property $U\left(t\right)U{\left(t\right)}^{†}=U_{0}U_{0}^{†}=I_{H⊗F}$ for all t⩾0. The system variables at time t⩾0, as operators on the system–field space H⊗F, are the images

(7) $$X_{k}\left(t\right)=j_{t}\left(X_{k}\left(0\right)\right),\;k=1,\ldots ,n,$$

of their initial values under the quantum stochastic flow $j_{t}$, which maps a system operator $\sigma_{0}$ on the initial space H to the operator

(8) $$\sigma \left(t\right):=j_{t}\left(\sigma_{0}\right)=U{\left(t\right)}^{†}\left(\sigma_{0}⊗I_{F}\right)U\left(t\right)$$

on $H_{t}$ in (5). The resulting quantum adapted process σ satisfies the following Hudson–Parthasarathy QSDE [23,24]:

(9) $$d\sigma =G\left(\sigma \right)dt-i\left[\sigma ,L^{T}\right]dW,\;G\left(\sigma \right):=i\left[H,\sigma \right]+D\left(\sigma \right),$$

where use is made of the Hamiltonian and the coupling operators evolved by the flow $j_{t}$ from (8) as

(10) $$H\left(t\right)=j_{t}\left(H_{0}\right),\;L\left(t\right)=j_{t}\left(L_{0}\right):={\left(j_{t}\left(L_{k}\left(0\right)\right)\right)}_{1⩽k⩽m}.$$

Note that the flow acts on vectors and matrices of operators in an entry-wise fashion. Furthermore, D in (9) is the decoherence superoperator, which acts on σ(t) as

(11) $$D\left(\sigma \right):=\frac{1}{2}\left(L^{T}\Omega \left[\sigma ,L\right]+\left[L^{T},\sigma \right]\Omega L\right)=-\left[\sigma ,L^{T}\right]\Omega L-\frac{1}{2}\left[L^{T}\Omega L,\sigma \right].$$

The second equality in (11) is applicable to the case where σ is a vector of operators on which the superoperator D acts entry-wise. The superoperator G in (9) is the Gorini–Kossakowski–Sudarshan–Lindblad (GKSL) generator [45,46], which is a quantum counterpart of the infinitesimal generators of classical Markov processes [26]. The identity $(\sigma_{0}⊗I_{F})U=U\sigma$, which holds for system operators σ in view of (8) and the unitarity of U(t), allows the QSDE (6) to be represented in the Heisenberg picture by using (10) along with the commutativity (4) as

(12) $$dU=-U\left(i\left(Hdt+L^{T}dW\right)+\frac{1}{2}L^{T}\Omega Ldt\right).$$

In application to the vector X(t) of system variables, the quantum stochastic flow $j_{t}$ acts entry-wise as

(13) $$X\left(t\right):=j_{t}\left(X_{0}\right)=U{\left(t\right)}^{†}\left(X_{0}⊗I_{F}\right)U\left(t\right)$$

in accordance with (7) and (8), and so the corresponding QSDE (9) can be represented in vector–matrix form:

(14) $$dX=Fdt+GdW,\;\;F:=G\left(X\right),\;\;G:=-i\left[X,L^{T}\right],$$

where the *n*-dimensional drift vector $F={\left(G\left(X_{j}\right)\right)}_{1⩽j⩽n}$ and the dispersion (n×m) matrix $G=-i{\left(\left[X_{j},L_{k}\right]\right)}_{1⩽j⩽n,1⩽k⩽m}$ consist of time-varying self-adjoint operators on H⊗F.

The interaction of the system with the input field *W* produces the output fields $Y_{1}\left(t\right),\ldots ,Y_{m}\left(t\right)$ assembled into a vector

(15) $$Y\left(t\right):={\left(Y_{k}\left(t\right)\right)}_{1⩽k⩽m}=U{\left(t\right)}^{†}\left(I_{H}⊗W\left(t\right)\right)U\left(t\right),$$

where the system–field unitary evolution is applied to the current input field variables, reflecting the innovation nature of the quantum Wiener process and the continuous tensor product structure of the Fock space mentioned above. The output field *Y* satisfies the QSDE

(16) dY=2JLdt+dW,

where the matrix *J* is given by (2) and *L* is the vector of the system–field coupling operators from (10). The system–field interaction makes the output field *Y* different from the input field *W* only through the drift vector 2JL in (16).

The common unitary evolution in (13) and (15) preserves the commutativity between the system variables and the output field variables over the course of time t⩾0:

(17) $$\left[X\left(t\right),Y{\left(t\right)}^{T}\right]=U{\left(t\right)}^{†}\left[X_{0}⊗I_{F},I_{H}⊗W{\left(t\right)}^{T}\right]U\left(t\right)=0,$$

where the entries of $X_{0}$, W(t) commute as operators on different spaces H, F. For a similar reason, the output field *Y* inherits the CCR matrix *J* from the input quantum Wiener process *W*:

(18) $$\begin{array}{cc} \left[Y\left(t\right),Y{\left(t\right)}^{T}\right] & =U{\left(t\right)}^{†}\left[I_{H}⊗W\left(t\right),I_{H}⊗W{\left(t\right)}^{T}\right]U\left(t\right)\\ & =2itJU{\left(t\right)}^{†}I_{H⊗F}U\left(t\right)=2itJ, \end{array}$$

where (3) is used with s=t. By means of (1) and (2), the relation (18) can also be established as a corollary of the property that *Y* in (16) inherits the quantum Ito matrix Ω from *W* as $dYdY^{T}=dWdW^{T}=\Omega dt$.

In view of (6) (see also (12)), the quantum stochastic flow $j_{t}$ in (8) depends on the system Hamiltonian $H_{0}$ and the system–field coupling operators in $L_{0}$. In turn, $H_{0}$ and $L_{0}$ are usually functions of the initial system variables $X_{1}\left(0\right),\ldots ,X_{n}\left(0\right)$, such as polynomials or Weyl quantization integrals [27] (see also Equation (32) in [47]). The dependence of $H_{0}$, $L_{0}$ on X(0) is inherited by *H*, *L* as functions of *X* and, along with a given commutation structure of the system variables, specifies a particular form of the resulting QSDEs (14) and (16), thereby influencing their tractability.

## 3. Open Quantum Harmonic Oscillators with Parametric Dependence

An important class of quantum stochastic systems is provided by multimode open quantum harmonic oscillators (OQHOs) [18], which have an even number *n* of system variables $X_{1},\ldots ,X_{n}$ (for example, consisting of $\frac{n}{2}$ conjugate position–momentum pairs [28]) satisfying the Heisenberg CCRs

(19) $$[X\left(t\right),X{\left(t\right)}^{T}]=2i\Theta$$

on a dense domain in H⊗F, where the CCR matrix $\Theta \in A_{n}$ is identified with $\Theta ⊗I_{H⊗F}$ and remains unchanged over the course of time t⩾0. The Hamiltonian *H* of the OQHO is a quadratic function and the system–field coupling operators $L_{1},\ldots ,L_{m}$ in (10) are linear functions of the system variables:

(20) $$H=\frac{1}{2}X^{T}RX,\;L=NX,$$

which are parameterized by a real symmetric *energy matrix R* of order *n* (we denote the subspace of such matrices by $S_{n}$) and a *coupling matrix* $N\in R^{m\times n}$. In this case, the QSDEs (14) and (16) take the form

(21) dX=AXdt+BdW,dY=CXdt+dW,

where the drifts F=AX and 2JL=CX depend linearly on the system variables $X_{1},\ldots ,X_{n}$, with $A\in R^{n\times n}$ and $C\in R^{m\times n}$ and the dispersion matrices $G=B\in R^{n\times m}$ and $I_{m}$ being constant real matrices. This linearity makes several of the dynamic properties of (21) similar to those of a classical linear stochastic system with a state-space realization quadruple $\left(A,B,C,I_{m}\right)$ and the corresponding $C^{m\times m}$-valued transfer function *F* on the complex plane:

(22) $$F\left(s\right):=C{\left(sI_{n}-A\right)}^{-1}B+I_{m},\;s\in C,$$

allowing for application of transfer function techniques [48]. However, in addition to the noncommutative nature of quantum variables, the matrices of coefficients of these QSDEs have a specific parameterization in terms of the energy, coupling, and CCR matrices:

(23) $$A=2\Theta \left(R+N^{T}JN\right),\;B=2\Theta N^{T},\;C=2JN.$$

Because the energy matrix *R* is symmetric and the CCR matrices Θ and *J* are antisymmetric, the matrices *A*, *B* and *C* satisfy

(24) $$A\Theta +\Theta A^{T}+BJB^{T}=0,\;\Theta C^{T}+BJ=0.$$

These equalities pertain to the fulfillment of the CCRs (19) and the commutativity (17) at any moment of time, and provide necessary and sufficient conditions for the physical realizability (PR) [21,22] of the linear QSDEs (21) as an OQHO with the CCR matrix Θ for the system variables. The first equality in (24) has the structure of an algebraic Lyapunov equation (ALE) with respect to Θ, which has a unique solution if and only if the Kronecker sum $A⊕A:=I_{n}⊗A+A⊗I_{n}$ is a nonsingular matrix, that is, no two eigenvalues of *A* are centrally symmetric about the origin in C. The latter condition holds, for example, when the matrix *A* is Hurwitz. For more general open quantum systems (such as anharmonic oscillators, for which the dynamic variables satisfy the CCRs (19) even though the Hamiltonian and the coupling operators are not necessarily quadratic and linear functions of the system variables), the CCR preservation is secured by the unitary evolution of the system variables in (13).

By analogy with the state-space realizations of transfer functions in classical linear systems theory [36,37], we use the *input–output map* for the OQHO (21) with the matrix quadruple $\left(A,B,C,I_{m}\right)$:

(25) $$S_{A,B,C,I_{m}}:\left(X_{0},W\right)\mapsto Y.$$

Now, supposing that the energy and coupling matrices *R* and *N*, which specify the Hamiltonian and the coupling operators in (20), depend smoothly on an auxiliary parameter ϵ∈R (while the quantum Wiener process *W* is independent of ϵ), then so do the matrices *A*, *B*, and *C* in (23) and the system and output variables which comprise the vectors X(t) and Y(t). Here, the differentiability of *X*, *Y* is understood in the weak sense. The corresponding partial derivatives

(26) $$X{\left(t\right)}^{\prime }:=\partial_{\epsilon }X\left(t\right),\;Y{\left(t\right)}^{\prime }:=\partial_{\epsilon }Y\left(t\right)$$

at any t⩾0 give rise to adapted quantum processes with self-adjoint operator-valued entries satisfying the QSDEs

(27) $$dX^{\prime }=\left(A^{\prime }X+AX^{\prime }\right)dt+B^{\prime }dW,\;dY^{\prime }=\left(C^{\prime }X+CX^{\prime }\right)dt,$$

the second of which is in fact an ODE ${\left(Y^{\prime }\right)}^{{}^{\cdot }}=C^{\prime }X+CX^{\prime }$ involving the time derivative $\dot{\left(\;\right)}$ of $Y^{\prime }$, with zero initial conditions $X_{0}^{\prime }=0$, $Y_{0}^{\prime }=0$, because $X_{0}$ and $Y_{0}$ do not depend on ϵ. Here,

(28) $$A^{\prime }=2\Theta \left(R^{\prime }+N^{\prime T}JN+N^{T}JN^{\prime }\right),\;B^{\prime }=2\Theta N^{\prime T},\;C^{\prime }=2JN^{\prime }$$

are the derivatives of the matrices *A*, *B* and *C* from (23) with respect to the parameter ϵ satisfying the relations

(29) $$A^{\prime }\Theta +\Theta A^{\prime T}+B^{\prime }JB^{T}+BJB^{\prime T}=0,\;\Theta C^{\prime T}+B^{\prime }J=0,$$

which are obtained by differentiating the PR conditions (24) while the CCR matrices Θ and *J* remain constant. Because the matrices Θ and *J* are antisymmetric, whereby $\Theta A^{\prime T}+BJB^{\prime T}=-{\left(A^{\prime }\Theta +B^{\prime }JB^{T}\right)}^{T}$, the first equality in (29) implies that

(30) $$A^{\prime }\Theta +B^{\prime }JB^{T}\in S_{n}.$$

By assembling the system variables and their parametric derivatives from (26) to an augmented vector

(31) $$S:=\left[\begin{array}{c} X\\ X^{\prime } \end{array}\right],$$

a combination of the QSDEs (21) and (27) allows the parametric derivative of the map (25) to be represented as the input–output map

(32) $$S_{A,B,C,I_{m}}^{\prime }=S_{A,B,C,0}:\left(X_{0},W\right)\mapsto Y^{\prime }$$

associated with

(33) $$dS=ASdt+BdW,\;{\left(Y^{\prime }\right)}^{{}^{\cdot }}=CS.$$

Here, in view of $X_{0}^{\prime }=0$, the initial condition $S_{0}=\left[\begin{array}{c} X_{0}\\ 0 \end{array}\right]$ is identified with $X_{0}$, and the matrices $A\in R^{2n\times 2n}$, $B\in R^{2n\times m}$ and $C\in R^{m\times 2n}$ are given by

(34) $$A:=\left[\begin{array}{cc} A & 0\\ A^{\prime } & A \end{array}\right],\;B:=\left[\begin{array}{c} B\\ B^{\prime } \end{array}\right],\;C:=\left[\begin{array}{cc} C^{\prime } & C \end{array}\right].$$

The same can be obtained by using the transfer functions of the corresponding linear systems (including (22)) as

$$\begin{array}{cc} F{\left(s\right)}^{\prime } & =C^{\prime }{\left(sI_{n}-A\right)}^{-1}B+C{\left(sI_{n}-A\right)}^{-1}A^{\prime }{\left(sI_{n}-A\right)}^{-1}B+C{\left(sI_{n}-A\right)}^{-1}B^{\prime }\\ & =C\left[\begin{array}{cc} {\left(sI_{n}-A\right)}^{-1} & 0\\ {\left(sI_{n}-A\right)}^{-1}A^{\prime }{\left(sI_{n}-A\right)}^{-1} & {\left(sI_{n}-A\right)}^{-1} \end{array}\right]B=C{\left(sI_{2n}-A\right)}^{-1}B \end{array}$$

for any s∈C which is not an eigenvalue of *A*. Here, use is made of a particular case of the block matrix inverse formula [49]:

$${\left[\begin{array}{cc} \alpha & 0\\ \gamma & \beta \end{array}\right]}^{-1}=\left[\begin{array}{cc} \alpha^{-1} & 0\\ -\beta^{-1}\gamma \alpha^{-1} & \beta^{-1} \end{array}\right].$$

The block lower triangular structure of the dynamics matrix A in (32) and (34) is closely related to the Gateaux derivative of the matrix exponential $e^{A}$ in the direction $A^{\prime }$ (see for example [50]):

(35) $${\left(e^{A}\right)}^{\prime }=\left[\begin{array}{cc} 0 & I_{n} \end{array}\right]e^{A}\left[\begin{array}{c} I_{n}\\ 0 \end{array}\right]=\int_{0}^{1}e^{(1-s)A}A^{\prime }e^{sA}ds.$$

At any time t⩾0, both $X{\left(t\right)}^{\prime }$ and $Y{\left(t\right)}^{\prime }$ depend linearly on the matrices $R^{\prime }$ and $N^{\prime }$ from (28) in view of the following representation of the solutions of the QSDEs (27), which regards $A^{\prime }Xdt+B^{\prime }dW$ as a forcing term in the first of these QSDEs:

(36) $$X{\left(t\right)}^{\prime }=\int_{0}^{t}e^{(t-s)A}\left(A^{\prime }X\left(s\right)ds+B^{\prime }dW\left(s\right)\right),\;Y{\left(t\right)}^{\prime }=\int_{0}^{t}\left(C^{\prime }X\left(s\right)+CX{\left(s\right)}^{\prime }\right)ds,$$

where

(37) $$X\left(t\right)=e^{tA}X_{0}+\int_{0}^{t}e^{(t-s)A}BdW\left(s\right)$$

is the unperturbed solution of the first QSDE in (21) which does not depend on $R^{\prime }$ and $N^{\prime }$. In the case of linear QSDEs with coefficients that depend smoothly on parameters, the mean square differentiability of their solutions with respect to those parameters can be verified directly by using the closed form (37) under certain integrability conditions for the underlying quantum state (specified by a density operator ρ on the system–field space H⊗F) in terms of relevant moments of the system variables such as $E(X_{0}^{T}X_{0})<+\infty$. Here, Eξ:=Tr(ρξ) is the quantum expectation which extends entry-wise to vectors and matrices of quantum variables.

An additional insight into the structure of the process $X^{\prime }$ is provided by its cross-commutation relations with *X* and *W*. In particular, because $X^{\prime }$ inherits adaptedness from the underlying system variables, it commutes with the Ito increments of the input field *W* in accordance with (4), and so does S in (31):

(38) $$\left[S,dW^{T}\right]=\left[\begin{array}{c} \left[X,dW^{T}\right]\\ {}[X^{\prime },dW^{T}] \end{array}\right]=0.$$

Furthermore, the differentiation of (19) in ϵ, combined with the identity $\left[\xi ,\eta^{T}\right]=-{\left[\eta ,\xi^{T}\right]}^{T}$ for vectors ξ and η of operators, leads to

(39) $$0=\left[X^{\prime },X^{T}\right]+\left[X,X^{\prime T}\right]=\left[X^{\prime },X^{T}\right]-{\left[X^{\prime },X^{T}\right]}^{T},$$

whereby the cross-commutation matrix $\left[X^{\prime },X^{T}\right]$ is symmetric, which holds regardless of a particular structure of the QSDEs (21) and remains valid for quantum anharmonic oscillators with nonlinear dynamics. This matrix evolves in time (with zero initial condition, as $X_{0}^{\prime }=0$) and has a steady-state value computed below.

**Theorem** **1.** *Suppose that the matrix A of the OQHO (21) in (23) is Hurwitz. Then, there exist the following limits*

(40) $$\lim_{t\to +\infty }\left[X{\left(t\right)}^{\prime },X{\left(t\right)}^{T}\right]=2i\Theta_{21},\;\lim_{t\to +\infty }\left[X{\left(t\right)}^{\prime },X{\left(t\right)}^{\prime T}\right]=2i\Theta_{22},$$

*where the matrices $\Theta_{21}\in S_{n}$ and $\Theta_{22}\in A_{n}$ are found as unique solutions of the ALEs*

(41) $$\begin{array}{cc} A\Theta_{21}+\Theta_{21}A^{T}+A^{\prime }\Theta +B^{\prime }JB^{T} & =0, \end{array}$$

(42) $$\begin{array}{cc} A\Theta_{22}+\Theta_{22}A^{T}-A^{\prime }\Theta_{21}+\Theta_{21}A^{\prime T}+B^{\prime }JB^{\prime T} & =0. \end{array}$$

**Proof.**  In view of the CCRs (19), the commutator matrix for the vector S in (31) is organized as

(43) $$\Xi :=\left[S,S^{T}\right]=\left[\begin{array}{cc} \left[X,X^{T}\right] & \left[X,X^{\prime T}\right]\\ {}[X^{\prime },X^{T}] & \left[X^{\prime },X^{\prime T}\right] \end{array}\right]=\left[\begin{array}{cc} 2i\Theta & \Xi_{12}\\ \Xi_{21} & \Xi_{22} \end{array}\right],$$

where the blocks $\Xi_{21}:=\left[X^{\prime },X^{T}\right]=-\Xi_{12}^{T}$, $\Xi_{12}:=\left[X,X^{\prime T}\right]$ and $\Xi_{22}:=\left[X^{\prime },X^{\prime T}\right]$ consist of time-varying skew self-adjoint operators on the system–field space H⊗F. By using the quantum Ito lemma [23,24] and the bilinearity of the commutator along with the QSDE (33) and the commutativity (38), it follows that (43) satisfies the QSDE

$$\begin{array}{cc} d\Xi & =\left[dS,S^{T}\right]+\left[S,dS^{T}\right]+\left[dS,dS^{T}\right]\\ & =\left[ASdt+BdW,S^{T}\right]+\left[S,S^{T}A^{T}dt+dW^{T}B^{T}\right]+\left[BdW,dW^{T}B^{T}\right]\\ & =A\left[S,S^{T}\right]dt+B\left[dW,S^{T}\right]+\left[S,S^{T}\right]A^{T}dt+\left[S,dW^{T}\right]B^{T}+B\left[dW,dW^{T}\right]B^{T}\\ & =(A\Xi +\Xi A^{T}+2iBJB^{T})dt, \end{array}$$

which, similarly to Equation (56) in [21], reduces to the ODE

(44) $$\dot{\Xi }=A\Xi +\Xi A^{T}+2iBJB^{T}$$

with the initial condition

(45) $$\Xi_{0}=\left[\begin{array}{cc} 2i\Theta & 0\\ 0 & 0 \end{array}\right]$$

in view of $X_{0}^{\prime }=0$. Hence, at any time t⩾0 the matrix Ξ(t) is an imaginary antisymmetric matrix which can be represented as

(46) $$\Xi \left(t\right)=2i\left[\begin{array}{cc} \Theta & \Theta_{12}\left(t\right)\\ \Theta_{21}\left(t\right) & \Theta_{22}\left(t\right) \end{array}\right]$$

with time-varying matrices $\Theta_{12}\left(t\right)=-\Theta_{21}{\left(t\right)}^{T}\in R^{n\times n}$ and $\Theta_{22}\left(t\right)\in A_{n}$. Substitution of (46) into (44) followed by using (34) leads to the Lyapunov ODEs

(47) $$\begin{array}{cc} {\dot{\Theta }}_{21} & =A\Theta_{21}+\Theta_{21}A^{T}+A^{\prime }\Theta +B^{\prime }JB^{T}, \end{array}$$

(48) $$\begin{array}{cc} {\dot{\Theta }}_{22} & =A\Theta_{22}+\Theta_{22}A^{T}+A^{\prime }\Theta_{12}+\Theta_{21}A^{\prime T}+B^{\prime }JB^{\prime T}, \end{array}$$

with zero initial conditions $\Theta_{21}\left(0\right)=0$ and $\Theta_{22}\left(0\right)=0$ in accordance with the corresponding blocks in (45). Now, the matrix $\Theta_{21}\left(t\right)$ is symmetric at any time t⩾0 due to (39). This follows from (47) in view of the symmetry (30). Hence, $\Theta_{12}=-\Theta_{21}$, and the ODE (48) takes the form

(49) $${\dot{\Theta }}_{22}=A\Theta_{22}+\Theta_{22}A^{T}-A^{\prime }\Theta_{21}+\Theta_{21}A^{\prime T}+B^{\prime }JB^{\prime T}.$$

If the matrix *A* is Hurwitz, then the solutions of the ODEs (47) and (49) converge to their unique steady-state values which (slightly abusing notation) satisfy the ALEs (41) and (42) and specify the limits (40). □

The relations (28)–(36) and Theorem 1 provide infinitesimal perturbation analysis for sensitivity of the internal and output variables of the OQHO to the matrices *R* and *N*. Under the perturbations of *R* and *N*, the Hamiltonian *H* and the coupling operators in *L* given by (20) remain in the corresponding classes of quadratic and linear functions of the system variables. Accordingly, the above analysis is not applicable to more general perturbations, for example, higher order polynomials of the system variables, and is restricted to linear QSDEs, meaning that an alternative approach is needed in the general case.

## 4. Transverse Hamiltonian

For the general quantum stochastic system described in Section 2 and governed by (14) and (16), we will now consider its response to arbitrary perturbations in the system Hamiltonian $H_{0}$ and the system–field coupling operators in $L_{0}$. More precisely, suppose that they are perturbed in directions $K_{0}$ and $M_{0}$ as

(50) $$H_{0}\mapsto H_{0}+\epsilon K_{0},\;L_{0}\mapsto L_{0}+\epsilon M_{0}.$$

Here, $K_{0}$ and the entries of the *m*-dimensional vector $M_{0}$ are self-adjoint operators on the initial system space H, while ϵ is a small real-valued parameter as before, and hence, $K_{0}=H_{0}^{\prime }$ and $M_{0}=L_{0}^{\prime }$. In what follows, the derivative ${\left(\cdot \right)}^{\prime }:=\partial_{\epsilon }\left(\cdot \right)$ is taken at ϵ=0. The perturbations $K_{0}$ and $M_{0}$ in (50) are assumed to be functions of the initial system variables $X_{1}\left(0\right),\ldots ,X_{n}\left(0\right)$, and these dependencies describe

(51) $$K\left(t\right):=j_{t}\left(K_{0}\right),\;M\left(t\right):=j_{t}\left(M_{0}\right)$$

as functions of X(t) under the *unperturbed* flow (8). For example, in the case of OQHOs considered in Section 3, the perturbations *K* and *M*, which are caused by the perturbations in the energy and coupling matrices *R* and *N*, inherit the structure of the Hamiltonian as a quadratic function and the coupling operators as linear functions of the system variables in (20), respectively:

(52) $$K=\frac{1}{2}X^{T}R^{\prime }X,\;M=N^{\prime }X.$$

Returning to the general case, at this stage we avoid technical assumptions regarding $K_{0}$ and $M_{0}$ in (50); thus, the calculations carried out below for arbitrary perturbations should be regarded as formal. Because the operators $H_{0}$ and $L_{0}$ completely specify the dynamics of the unitary operator U(t) in (6), which determines the evolution of the system and output field variables, the response of the latter to the perturbations (50) of $H_{0}$ and $L_{0}$ reduces to that of U(t). Therefore, the propagation of the initial perturbations $K_{0}$ and $M_{0}$ of the operators $H_{0}$ and $L_{0}$ through the subsequent unitary system–field evolution can be described in terms of the operator

(53) $$V\left(t\right):=U{\left(t\right)}^{\prime },$$

which satisfies $V_{0}=0$, as $U_{0}=I_{H⊗F}$ does not depend on ϵ. The smoothness of dependence of U(t) on the parameter ϵ is analogous to the corresponding property of solutions of classical SDEs (under suitable regularity conditions [26,51] for their drift and dispersion) and holds at least in the case of linear QSDEs discussed in Section 3. The following theorem is closely related to Stone’s theorem on generators of one-parameter unitary groups [52].

**Theorem** **2.** *For any time t⩾0, the operator V(t) in (53), associated with the unitary evolution U(t) from (6), can be represented as*

(54) V(t)=−iU(t)Q(t).

*Here, Q(t) is a self-adjoint operator on the system–field space H⊗F, which satisfies the zero initial condition $Q_{0}=0$ and is governed by the QSDE*

(55) dQ=(K−Im(LTΩM))dt+MTdW,

*where the imaginary part Im(·) is extended to operators and matrices of operators as Imμ:=12i(μ−μ#), with ${\left(\cdot \right)}^{\#}$ denoting the entry-wise operator adjoint. Furthermore, Q(t) depends linearly on the initial perturbations $K_{0}$ and $M_{0}$ of the Hamiltonian and coupling operators in (50) through their unperturbed evolutions in (51).*

**Proof.**  The differentiation of both sides of the unitarity relation $U{\left(t\right)}^{†}U\left(t\right)=I_{H⊗F}$ with respect to the parameter ϵ at ϵ=0 leads to $V^{†}U+U^{†}V={\left(U^{†}V\right)}^{†}+U^{†}V=0$, which implies self-adjointness of the operator

(56) $$Q\left(t\right):=iU{\left(t\right)}^{†}V\left(t\right),$$

establishing (54). Now, consider the time evolution of Q(t). To this end, the differentiation of (6) with respect to ϵ yields

(57) dV=−(i(K0dt+M0TdW)+Re(L0TΩM0)dt)U−i(H0dt+L0TdW)+12L0TΩL0dtV,

where the real part Re(·) is extended to operators and matrices of operators as Reμ:=12(μ+μ#). By left-multiplying both sides of (57) by $U^{†}$ and recalling (56), it follows that

(58) U†dV=−i(Kdt+MTdW)−Re(LTΩM)dt+i2LTΩLdt−Hdt−LTdWQ=−iK−Re(LTΩM)+i2LTΩL−HQdt−(LQ+iM)TdW,

where use is made of the evolved perturbations in (51). By a similar reasoning, a combination of (12) with (56) leads to

(59) $$\begin{array}{cc} (dU^{†})V & =\left(i\left(Hdt+L^{T}dW\right)-\frac{1}{2}L^{T}\Omega Ldt\right)U^{†}V\\ & =\left(Hdt+L^{T}dW+\frac{i}{2}L^{T}\Omega Ldt\right)Q, \end{array}$$

(60) $$\begin{array}{cc} dU^{†}dV & =iL^{T}dWU^{†}dV=-iL^{T}dWdW^{T}\left(LQ+iM\right)\\ & =L^{T}\Omega \left(M-iLQ\right)dt, \end{array}$$

where use is also made of (58) along with the quantum Ito product rules [24], (1) and (4). It now follows from (56) and (58)–(60) that

dQ=i((dU†)V+U†dV+dU†dV)=(K+iLTΩM−iRe(LTΩM))dt+MTdW=(K−Im(LTΩM))dt+MTdW,

which establishes (55). The linear dependence of Q(t) on $K_{0}$ and $M_{0}$ follows from the integral representation

(61) Q(t)=∫0t((K(s)−Im(L(s)TΩM(s)))ds+M(s)TdW(s))

of the QSDE (55) and the property that the evolved perturbations *K* and *M* in (51) depend linearly on $K_{0}$ and $M_{0}$, respectively. □

In view of Theorem 2, for any fixed but otherwise arbitrary time t⩾0, the relation (54), represented as

$$U{\left(t\right)}^{\prime }=-iU\left(t\right)Q\left(t\right),$$

has the structure of isolated quantum dynamics in fictitious time ϵ, where Q(t) plays the role of a Hamiltonian pertaining to the perturbation of the unitary operator U(t). In order to reflect this property, we will refer to the time-varying operator *Q* as the *transverse Hamiltonian* associated with the perturbations *K* and *M* of the system Hamiltonian *H* and the system–field coupling operators in *L*. The computation of *Q* is illustrated by the following two examples.

*Example 1*

In the absence of perturbation of the system–field coupling, when the vector $M_{0}$ in (50) consists of zero operators (and hence, so does *M* in (51)), the transverse Hamiltonian in (61) reduces to

(62) $$Q\left(t\right)=\int_{0}^{t}K\left(s\right)ds.$$

Moreover, if the system is isolated, that is, L=0, then (6) reduces to the ODE $\dot{U}\left(t\right)=-iH_{0}U\left(t\right)$, which leads to

(63) $$U\left(t\right)=e^{-itH_{0}},$$

with the Hamiltonian being preserved in time: $H\left(t\right)=H_{0}$ for all t⩾0. In this case of isolated system dynamics, (62) takes the form

(64) $$Q\left(t\right)=\int_{0}^{t}e^{isH_{0}}K_{0}e^{-isH_{0}}ds=\int_{0}^{t}e^{is{\mathrm{ad}}_{H_{0}}}\left(K_{0}\right)ds=tE\left(it{\mathrm{ad}}_{H_{0}}\right)\left(K_{0}\right),$$

where ${\mathrm{ad}}_{H_{0}}\left(\cdot \right):=\left[H_{0},\cdot \right]$. Here, use is made of Hadamard’s lemma [28] along with an entire function $E\left(z\right):=\frac{e^{z}-1}{z}=\sum_{k=0}^{+\infty }\frac{z^{k}}{(k+1)!}$ of a complex variable (with E(0)=1 by continuity) which plays a role in the solution of nonhomogeneous linear ODEs with constant coefficients and constant forcing terms [50]. The Gateaux derivative (53) of (63) can be represented using an operator version of (35) as

(65) $$\begin{array}{cc} V(t) & =\left[\begin{array}{cc} 0 & I_{H} \end{array}\right]\exp\left(-it\left[\begin{array}{cc} H_{0} & 0\\ K_{0} & H_{0} \end{array}\right]\right)\left[\begin{array}{c} I_{H}\\ 0 \end{array}\right]\\ & =-it\int_{0}^{1}e^{i\left(s-1\right)tH_{0}}K_{0}e^{-istH_{0}}ds=-iU\left(t\right)\int_{0}^{t}e^{isH_{0}}K_{0}e^{-sH_{0}}ds, \end{array}$$

which provides an alternative verification of (54) for this particular case, with *Q* given by (64).

*Example 2*

For the OQHO of Section 3, substitution of the perturbations from (52) into (61) leads to the transverse Hamiltonian

(66) Q(t)=∫0t12X(s)TR′X(s)−Im(X(s)TNTΩN′X(s))ds+X(s)TN′TdW(s)=∫0t12X(s)TR′+i(NTΩN′−N′TΩN)X(s)ds+X(s)TN′TdW(s)=∫0t12X(s)TR′+N′TJN−NTJN′X(s)ds+X(s)TN′TdW(s)−〈NΘ,N′〉t

which depends linearly on the matrices $R^{\prime }$ and $N^{\prime }$ as well as, in a quadratic fashion, on the past history of the system variables. The latter are given by the unperturbed Equation (37). The last term $\langle N\Theta ,N^{\prime }\rangle t=-\mathrm{Tr}(\Theta N^{T}N^{\prime })t$ in (66) (with $\langle \alpha ,\beta \rangle :=\mathrm{Tr}(\alpha^{T}\beta )$ denoting the Frobenius inner product for real matrices) comes from the relation Im(i(NTΩN′−N′TΩN))=NTN′−N′TN, which follows from (1), and the identity $X^{T}\Upsilon X=i\langle \Upsilon ,\Theta \rangle$, which holds for any matrix $\Upsilon \in A_{n}$ in view of the CCRs (19).

## 5. Infinitesimal Perturbation Analysis of System Operators

Because the transverse Hamiltonian Q(t) in (56), (61) encodes the propagation of the initial perturbations of the Hamiltonian and coupling operators in (50) through the unitary system–field evolution over the time interval [0,t], it provides a tool for infinitesimal perturbation analysis of general system operators. The following theorem is concerned with an extended setting which, in addition to (50), allows for smooth dependence of a system operator $\sigma_{0}$ on the parameter ϵ, meaning that an appropriate infinitesimal perturbation in it is specified by an operator $\sigma_{0}^{\prime }$ on the initial space H, with $\sigma_{0}^{\prime }$ being a function of the initial system variables $X_{1}\left(0\right),\ldots ,X_{n}\left(0\right)$.

**Theorem** **3.** *For any self-adjoint system operator $\sigma_{0}$ on the initial space H, which smoothly depends on the same scalar parameter ϵ as in (50) and is evolved by the flow (8), the derivative of its evolved version σ(t) with respect to ϵ can be represented as*

(67) $$\tau \left(t\right):=\sigma {\left(t\right)}^{\prime }=j_{t}\left(\sigma_{0}^{\prime }\right)+\phi \left(t\right),\;\phi \left(t\right):=i\left[Q\left(t\right),\sigma \left(t\right)\right]$$

*at any time t⩾0, where Q(t) is the transverse Hamiltonian from Theorem 2. Here, the operator ϕ(t) satisfies the QSDE*

(68) $$d\phi =\left(i\left[Q,G\left(\sigma \right)\right]+\chi \left(\sigma \right)\right)dt+\left(\left[Q,\left[\sigma ,L^{T}\right]\right]-i\left[\sigma ,M^{T}\right]\right)dW,$$

*with zero initial condition $\phi_{0}=0$, where G is the unperturbed GKSL generator from (9) and χ is a linear superoperator given by*

(69) χ(σ):=i[K−Im(LTΩM),σ]−2Re([σ,LT]ΩM).

**Proof.**  By using the Leibniz product rule together with (8), (53), (54) and the self-adjointness of Q(t), it follows that

(70) $$\begin{array}{cc} \sigma^{\prime } & =U^{†}\left(\sigma_{0}^{\prime }⊗I_{F}\right)U+V^{†}\left(\sigma_{0}⊗I_{F}\right)U+U^{†}\left(\sigma_{0}⊗I_{F}\right)V\\ & =j_{t}\left(\sigma_{0}^{\prime }\right)+{\left(-iUQ\right)}^{†}\left(\sigma_{0}⊗I_{F}\right)U-iU^{†}\left(\sigma_{0}⊗I_{F}\right)UQ\\ & =j_{t}\left(\sigma_{0}^{\prime }\right)+iQU^{†}\left(\sigma_{0}⊗I_{F}\right)U-i\sigma Q=j_{t}\left(\sigma_{0}^{\prime }\right)+i\left[Q,\sigma \right], \end{array}$$

which establishes (67), with the initial condition $\varphi_{0}=0$ being inherited by ϕ from $Q_{0}=0$. We will now obtain a QSDE for the process ϕ. To this end, by combining the quantum Ito lemma with the bilinearity of the commutator (similarly to the proof of Theorem 1) and using the QSDEs (9) and (55), it follows that

(71) d[Q,σ]=[dQ,σ]+[Q,dσ]+[dQ,dσ]=[K−Im(LTΩM),σ]dt−[σ,MT]dW+[Q,G(σ)]dt−i[Q,[σ,LT]]dW−[MTdW,i[σ,LT]dW]=([K−Im(LTΩM),σ]+[Q,G(σ)]+2iIm(i[σ,LT]ΩM))dt−([σ,MT]+i[Q,[σ,LT]])dW=([K−Im(LTΩM),σ]+[Q,G(σ)]+2iRe([σ,LT]ΩM))dt−([σ,MT]+i[Q,[σ,LT]])dW.

Here, the quantum Ito product rules are applied together with the commutativity (4) between adapted processes and the Ito increments of the quantum Wiener process *W*. In the second and third of the equalities (71), use is also made of the relations $\left[\alpha ,\beta^{T}dW\right]=\left[\alpha ,\beta^{T}\right]dW$ and [αTdW,βTdW]=2iIm(αTΩβ)dt for appropriately dimensioned adapted processes α and β with self-adjoint operator-valued entries. These relations are combined with the identity Im(i[σ,LT]ΩM)=Re([σ,LT]ΩM) in the fourth equality of (71). The QSDE (68) is now obtained my multiplying both sides of (71) by *i* and using the superoperator χ from (69). □

As can be seen from (70), the process $j_{t}\left(\sigma_{0}^{\prime }\right)$ in (67) is the response of σ(t) to the initial perturbation $\sigma_{0}^{\prime }$ of the system operator under the unperturbed flow (8) and satisfies the QSDE (9):

(72) $$dj_{t}\left(\sigma_{0}^{\prime }\right)=G\left(j_{t}\left(\sigma_{0}^{\prime }\right)\right)dt-i\left[j_{t}\left(\sigma_{0}^{\prime }\right),L^{T}\right]dW.$$

In contrast to $j_{t}\left(\sigma_{0}^{\prime }\right)$, the operator ϕ(t) in (67) describes the response of the flow $j_{t}$ itself to the perturbations (50) of the Hamiltonian and coupling operators and will be referred to as the *derivative process* for the system operator σ. Accordingly, the term i[Q,G(σ)] in the drift of the QSDE (68) is the derivative process for G(σ). At any given time t⩾0, the last equality in (67) is organized as the right-hand side of the Heisenberg ODE (in fictitious time ϵ) of an isolated quantum system with the state space H⊗F and the Hamiltonian Q(t). While $j_{t}\left(\sigma_{0}^{\prime }\right)$ (evolved by the unperturbed flow) depends linearly on $\sigma_{0}^{\prime }$, the derivative process ϕ(t) depends linearly on the perturbations $K_{0}$ and $M_{0}$ of the Hamiltonian $H_{0}$ and the coupling operators in $L_{0}$ through the transverse Hamiltonian *Q* and the superoperator χ in (69).

In application to the vectors *X* and *L* of the system variables and the system–field coupling operators, the QSDEs (68) and (72) and the definition (69) lead to

(73) dX′=(i([K−Im(LTΩM),X]+[Q,F])+2Im(GΩM))dt+i([Q,G]−[X,MT])dW,

(74) dL′=(G(M)+i([K−Im(LTΩM),L]+[Q,G(L)])−2Re([L,LT]ΩM))dt+([Q,[L,LT]]−i[L,MT]−i[M,LT])dW,

where *F* and *G* are the unperturbed drift vector and the dispersion matrix from (14). In (73), use is also made of the absence $X_{0}^{\prime }=0$ of initial perturbations in the system variables, whereby $j_{t}\left(X_{0}^{\prime }\right)=0$ for any t⩾0. Furthermore, in (74) we have used the relations $j_{t}\left(L_{0}^{\prime }\right)=j_{t}\left(M_{0}\right)=M\left(t\right)$ in view of (50) and (51). Because the matrix *J* and the quantum Wiener process *W* do not depend on the parameter ϵ, the derivative of the output field *Y* of the system in (16) evolves according to the ODE

(75) $${\left(Y^{\prime }\right)}^{{}^{\cdot }}=2JL^{\prime },$$

where $L^{\prime }$ is governed by the QSDE (74).

In particular, for the OQHO from Section 3 with the perturbations in (52), the QSDEs (73)–(75) lead to the relations (27) and (28) which were obtained in Section 3 using more elementary techniques. However, the latter are limited to quadratic perturbations of the Hamiltonian and linear perturbations of the coupling operators in (52), whereas the transverse Hamiltonian approach allows the system response to be investigated for general perturbations of these operators. Therefore, this approach can be used for the development of optimality conditions in quantum control and filtering problems for larger classes of controllers and observers.

## 6. Sensitivity of Infinite-Horizon Quantum Averaged Functionals

Similarly to optimal control of classical time invariant stochastic systems [36,37], suppose that the infinite-horizon performance of the quantum system under consideration is described by a cost functional

(76) $$Z:=\lim_{t\to +\infty }EZ\left(t\right),$$

which (whenever it exists) leads to the same Cesaro limit $\lim_{t\to +\infty }\left(\frac{1}{t}\int_{0}^{t}EZ\left(s\right)ds\right)=Z$ and is to be minimized in optimal quantum control settings. Here, the quantum expectation E(·) is taken over the density operator

(77) ρ:=ϖ⊗υ,

which is the tensor product of the initial quantum state ϖ of the system on the space H and the vacuum state [24] υ on the Fock space F for the external bosonic fields. This expectation is applied in (76) to a quantum *criterion process Z*, specified as

(78) Z(t):=f(X(t),L(t))

using a function $f:R^{n+m}\to R$. The latter is extended to the noncommutative system variables and the coupling operators (with *L* being in a bijective correspondence with the drift vector 2JL of the output field in (16), as detJ≠0 in view of (2)) to make Z(t) a self-adjoint operator for any t⩾0. Such an operator-valued extension of *f* is straightforward in the case of polynomials and can be carried out through the Weyl quantization [27] for more general functions. In the coherent quantum linear–quadratic Gaussian (CQLQG) control and filtering problems [20,35,38], the function *f* in (78) is a positive semi-definite quadratic form, in which case the minimization of (76) provides an infinite-horizon mean square optimality criterion.

Now, if the Hamiltonian and coupling operators of the quantum system are perturbed according to (50), then application of the transverse Hamiltonian *Q* from Theorems 2 and 3 leads to

(79) $${\left(EZ\left(t\right)\right)}^{\prime }=E\left(j_{t}\left(Z_{0}^{\prime }\right)+\phi \left(t\right)\right),\;\phi \left(t\right):=i\left[Q\left(t\right),Z\left(t\right)\right],$$

where ϕ is the derivative process for *Z*. Note that despite the equality $X_{0}^{\prime }=0$, the operator $Z_{0}^{\prime }$ can be nonzero due to the dependence of $Z_{0}=f\left(X_{0},L_{0}\right)$ in (78) on $L_{0}$ which is being perturbed. Assuming the existence and interchangeability of appropriate limits, (79) leads to the following perturbation formula for the cost functional Z in (76):

(80) $$Z^{\prime }:=\lim_{t\to +\infty }\left(Ej_{t}\left(Z_{0}^{\prime }\right)+E\phi \left(t\right)\right).$$

The right-hand side of (80) is a linear functional of the perturbations $K_{0}$ and $M_{0}$, which describes the corresponding (formal) Gateaux derivative of Z in the direction $\left(K_{0},M_{0}\right)$. Therefore, the quantum system is a stationary point of the performance criterion (76) with respect to a subspace T of perturbations $\left(K_{0},M_{0}\right)$ in (50) if T is contained by the null space of the linear functional $Z^{\prime }$ in (80):

(81) $$T\subset \ker Z^{\prime }.$$

This inclusion provides a first-order necessary condition of optimality in the quantum control problem of minimizing (76) over a manifold of the Hamiltonian and coupling operators with the local tangent space T.

While $\lim_{t\to +\infty }Ej_{t}\left(Z_{0}^{\prime }\right)$ in (80) reduces to averaging over the invariant quantum state of the unperturbed system (provided certain integrability conditions are satisfied together with the existence of and weak convergence to the invariant state), the computation of $\lim_{t\to +\infty }E\phi \left(t\right)$ is less straightforward. Due to the product structure (77) of the system–field state ρ (with the external fields being in the vacuum state υ), the martingale part $(\left[Q,\left[Z,L^{T}\right]\right]-i\left[Z,M^{T}\right])dW$ of the QSDE (68) with σ:=Z does not contribute to the time derivative

(82) $${\left(E\phi \right)}^{{}^{\cdot }}=E\psi +E\chi \left(Z\right),\;\psi :=i\left[Q,G\left(Z\right)\right],$$

where ψ is the derivative process for G(Z) and the superoperator χ from (69) is applied to the criterion process *Z* in (78).

The relation (82) is a complicated integro-differential equation (IDE). Nevertheless, this IDE admits an efficient solution, for example, in the case when the system is an OQHO, and the function *f* in (78) is a polynomial. In this case, due to the structure of the GKSL generator of the OQHO, G(Z) is a polynomial in the system variables of the same degree, leading to a linear relation (with constant coefficients) between the derivative processes ϕ and ψ and to algebraic closedness in the IDE (82). Therefore, for stable OQHOs (that is, with a Hurwitz matrix *A*) and a polynomial criterion process *Z*, the computation of $Z^{\prime }$ and verification of the stationarity condition (81) reduce to averaging over the unperturbed invariant state, which is unique and Gaussian [34]. These calculations are exemplified below.

*Example 3*

Consider the OQHO from Section 3 described by (19)–(23) with a Hurwitz matrix *A*. Suppose the criterion process *Z* in (78) is a quadratic polynomial of the system variables $X_{1},\ldots ,X_{n}$ and the system–field coupling operators $L_{1},\ldots ,L_{m}$:

(83) $$Z:=\frac{1}{2}\left[\begin{array}{cc} X^{T} & L^{T} \end{array}\right]\Pi \left[\begin{array}{c} X\\ L \end{array}\right]=\frac{1}{2}X^{T}PX,\;\Pi :=\left[\begin{array}{cc} \Pi_{11} & \Pi_{12}\\ \Pi_{21} & \Pi_{22} \end{array}\right],$$

where $\Pi \in S_{n+m}^{+}$ is a given weighting matrix partitioned into blocks $\Pi_{11}\in S_{n}^{+}$, $\Pi_{22}\in S_{m}^{+}$ and $\Pi_{12}=\Pi_{21}^{T}\in R^{n\times m}$, and

(84) $$P:=\Pi_{11}+\Pi_{12}N+N^{T}\Pi_{21}+N^{T}\Pi_{22}N\in S_{n}^{+}$$

is an auxiliary matrix which involves the coupling matrix *N* from (20). Here, $S_{r}^{+}$ denotes the set of real positive semi-definite symmetric matrices of order *r*. Then, in view of the relation $X_{0}^{\prime }=0$, it follows from (83) and (52) that

(85) jt(Z0′)=Re((Π21X+Π22L)TM)=Re(XT(Π12+NTΠ22)M).

The second equality in (83) allows the quantum average of the corresponding derivative process ϕ in (79) to be represented as

(86) Eϕ=12〈P,Υ〉,Υ:=iE[Q,Ξ],Ξ:=Re(XXT)=XXT−iΘ.

Here, Υ is the expectation of the derivative process i[Q,Ξ] for Ξ and, as such, satisfies the following IDE, similar to (82):

(87) $$\dot{\Upsilon }=iE\left[Q,G\left(\Xi \right)\right]+E\chi \left(\Xi \right),$$

where the superoperator χ from (69) is applied to Ξ entry-wise as

(88) χ(XjXk)=i[K−Im(LTΩM),XjXk]−2Re([XjXk,LT]ΩM)=i[K−Im(XTNTΩM),XjXk]+4Im((XkΘj•+XjΘk•)NTΩM),

with $\Theta_{\ell •}$ denoting the *ℓ*th row of the CCR matrix Θ. By substituting the Hamiltonian and coupling operators of the OQHO from (20) into the GKSL generator G in (9) and (11) and using the CCRs (19) together with the state-space matrices (23), it follows that

(89) $$G\left(\Xi \right)=A\Xi +\Xi A^{T}+BB^{T}.$$

Substitution of (89) into (87) reduces the IDE to a Lyapunov ODE:

(90) $$\dot{\Upsilon }=iE\left[Q,A\Xi +\Xi A^{T}+BB^{T}\right]+E\chi \left(\Xi \right)=A\Upsilon +\Upsilon A^{T}+E\chi \left(\Xi \right).$$

Because the matrix *A* is Hurwitz, the unperturbed OQHO has an invariant state which is Gaussian [34] with zero mean EX=0 and a complex positive semi-definite Hermitian covariance matrix $E(XX^{T})=\Sigma +i\Theta$ of order *n*, for which the real part $\Sigma \in S_{n}^{+}$ is a unique solution of the ALE

(91) $$A\Sigma +\Sigma A^{T}+BB^{T}=0.$$

Therefore, under appropriate integrability conditions for χ(Ξ), the solution of the Lyapunov ODE (90) has a limit $\Upsilon_{\infty }:=\lim_{t\to +\infty }\Upsilon \left(t\right)$, which is a unique solution of the ALE

(92) $$A\Upsilon_{\infty }+\Upsilon_{\infty }A^{T}+\lim_{t\to +\infty }E\chi \left(\Xi \right)=0,$$

where $\lim_{t\to +\infty }E\chi \left(\Xi \right)$ can be computed by averaging χ(Ξ) over the invariant Gaussian state of the OQHO. Therefore, by assembling (85) and (86) into (80), it follows that

(93) Z′=limt→+∞ERe(XT(Π12+NTΠ22)M)+12〈P,Υ∞〉,

where the limit reduces to averaging over the invariant Gaussian state and is expressed in terms of $E(XM^{T})$ as

(94) ERe(XT(Π12+NTΠ22)M)=〈Π12+NTΠ22,ReE(XMT)〉.

The relations (84), (88) and (92)–(94) allow the Gateaux derivative $Z^{\prime }$ of the quadratic cost functional (76), specified by (83), to be computed through mixed moments of the system variables *X* and the perturbations *K* and *M* over the invariant zero-mean Gaussian quantum state, the covariance matrix of which can be found from (91). In particular, if *K* and *M* are polynomials of the system variables $X_{1},\ldots ,X_{n}$, then these moments can be calculated in terms of the covariances by using the Isserlis–Wick theorem [53]. Alternatively, the perturbations *K* and *M* can be trigonometric polynomials, that is, linear combinations of unitary Weyl operators [27]

(95) $$W_{u}:=e^{iu^{T}X}=W_{-u}^{†},\;u\in R^{n},$$

associated with the system variables, or more generally represented as the Weyl quantization integrals

(96) $$K:=\int_{R^{n}}W_{u}\alpha \left(du\right),\;M:=\int_{R^{n}}W_{u}\beta \left(du\right),$$

similar to those in Equation (32) of [47]. Here, α and β are countably additive measures of finite variation on the σ-algebra $B^{n}$ of Borel subsets of $R^{n}$, which take values in C and $C^{m}$, respectively, and satisfy the Hermitian property $\bar{\alpha (S)}=\alpha \left(-S\right)$ and $\bar{\beta (S)}=\beta \left(-S\right)$ (where $\bar{\left(\cdot \right)}$ is the complex conjugate) for any $S\in B^{n}$, ensuring that *K* and the entries of *M* in (96) are self-adjoint operators in view of the second equality in (95). Now, the Weyl CCRs $W_{u+v}=e^{iu^{T}\Theta v}W_{u}W_{v}$ for all $u,v\in R^{n}$ (with their infinitesimal Heisenberg form given by (19)) imply that $\partial_{u}W_{u}=\partial_{v}e^{iv^{T}\left(X+\Theta u\right)}{|}_{v=0}W_{u}=i\left(X+\Theta u\right)W_{u}$, whereby

(97) $$XW_{u}=-\left(\Theta u+i\partial_{u}\right)W_{u}.$$

Because the quantum expectation commutes with the differential operator on the right-hand side of (97) and the quasi-characteristic function [54] of the invariant zero-mean Gaussian state is given by $EW_{u}=e^{-\frac{1}{2}{∥u∥}_{\Sigma }^{2}}$, where ${∥u∥}_{\Sigma }:=\sqrt{u^{T}\Sigma u}$ is a weighted Euclidean semi-norm of *u* specified by Σ, we have

(98) $$\begin{array}{cc} E(XW_{u}) & =-\left(\Theta u+i\partial_{u}\right)EW_{u}=-\left(\Theta u+i\partial_{u}\right)e^{-\frac{1}{2}{∥u∥}_{\Sigma }^{2}}\\ & =e^{-\frac{1}{2}{∥u∥}_{\Sigma }^{2}}\left(i\Sigma -\Theta \right)u,\;u\in R^{n}, \end{array}$$

which can also be obtained using quantum Price’s theorem [55]. A combination of the second equality from (96) with (98) leads to

(99) ReE(XMT):=Re∫RnE(XWu)β(du)T=∫Rne−12∥u∥Σ2Re((iΣ−Θ)uβ(du)T)=−∫Rne−12∥u∥Σ2(ΣuImβ(du)T+ΘuReβ(du)T).

The computation of the limit in (93) in the Weyl quantization framework can now be completed by substituting (99) into (94). In this framework, the matrix Eχ(Ξ), which is needed for finding $\Upsilon_{\infty }$ from the ALE (92) for the last term in (93), is computed by averaging (88) in a similar fashion.

## 7. Mean Square Optimal Coherent Quantum Filtering Problem

We will now demonstrate an application of the transverse Hamiltonian variational approach from Section 4, Section 5 and Section 6 to a quantum filtering problem for the open quantum system described in Section 2, referred to as a *quantum plant*. The plant has the Hamiltonian *H*, vector *L* of system–field coupling operators, input field *W*, vector *X* of internal variables, and output field *Y* governed by the QSDEs (14) and (16). Although the plant is not necessarily an OQHO, we assume that the plant variables $X_{1},\ldots ,X_{n}$ are organized as position–momentum pairs and satisfy the CCRs (19).

**Figure 1:**
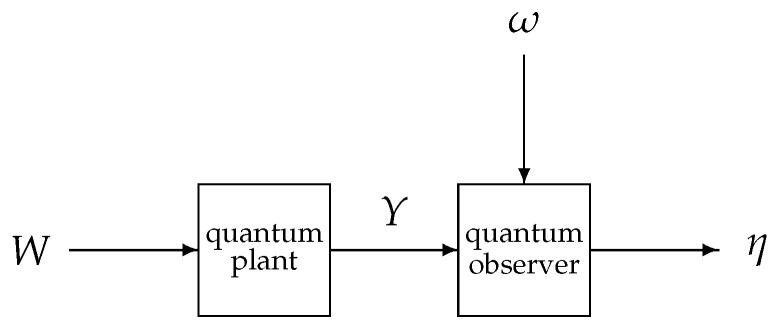
The series connection of a quantum plant with a coherent quantum observer, mediated by the plant output field *Y* and affected by the environment through the input quantum Wiener processes *W* and ω. The observer design objective is that the drift part of its output η has to approximate the plant variables of interest in a mean square optimal fashion.

Suppose that the quantum plant is cascaded in a measurement-free field-mediated fashion with another open quantum system, which plays the role of a *coherent quantum observer* and is driven by the plant output *Y* and another quantum Wiener process ω of an even dimension μ on a different symmetric Fock space F; see Figure 1.

The observer is endowed with its own initial Hilbert space H, and dynamic variables $\xi_{1}\left(t\right),\ldots ,\xi_{\nu }\left(t\right)$ with a CCR matrix $\Lambda \in A_{\nu }$ such that $[\xi \left(t\right),\xi {\left(t\right)}^{T}]=2i\Lambda$ for any t⩾0, similarly to (19), as well as an (m+μ)-dimensional output field η(t):

(100) $$\xi :={\left(\xi_{k}\right)}_{1⩽k⩽\nu },\;\omega :={\left(\omega_{k}\right)}_{1⩽k⩽\mu },\;\eta :={\left(\eta_{k}\right)}_{1⩽k⩽m+\mu }.$$

As the observer is driven by the plant output *Y* together with the quantum noise ω, over the course of time the observer output η acquires quantum statistical correlations with the plant variables and can be used for estimating the latter in a mean square optimal fashion as specified below. To this end, we denote the observer Hamiltonian by Γ and the vectors of operators of coupling of the observer with the plant output *Y* and the quantum Wiener process ω by

(101) $$\Phi :={\left(\Phi_{k}\right)}_{1⩽k⩽m},\;\Psi :={\left(\Psi_{k}\right)}_{1⩽k⩽\mu },$$

respectively. The Hamiltonian Γ and the coupling operators $\Phi_{1},\ldots ,\Phi_{m}$, $\Psi_{1},\ldots ,\Psi_{\mu }$ are functions of the dynamic variables $\xi_{1},\ldots ,\xi_{\nu }$ of the observer and hence (by the commutativity $[X,\xi^{T}]=0$) commute with the plant variables and functions thereof, including *H* and *L*. The plant and observer form a composite quantum stochastic system with a vector X of dynamic variables satisfying the CCRs

(102) $$\left[X,X^{T}\right]=2i\Theta ,\;\Theta :=\left[\begin{array}{cc} \Theta & 0\\ 0 & \Lambda \end{array}\right],\;X:=\left[\begin{array}{c} X\\ \xi \end{array}\right]$$

and driven by an augmented quantum Wiener process W with the following Ito table:

(103) $$dWdW^{T}=\Omega dt,\;\Omega :=\left[\begin{array}{cc} \Omega & 0\\ 0 & ℧ \end{array}\right]=I_{m+\mu }+iJ,\;J:=I_{(m+\mu )/2}⊗J,\;W:=\left[\begin{array}{c} W\\ \omega \end{array}\right].$$

Here, ℧ is the Ito matrix of the quantum Wiener process ω of the observer, which is defined similarly to Ω from (1) and (2) as

(104) $$d\omega d\omega^{T}=℧dt,\;℧:=I_{\mu }+iI_{\mu /2}⊗J.$$

The Ito matrix Ω of the process W in (103) is block-diagonal due to the commutativity $[W,\omega^{T}]=0$ and the statistical independence between the quantum Wiener processes *W* and ω, which act on the different Fock spaces F and F and are assumed to be in the appropriate vacuum states. The quantum feedback network formalism [7] allows the Hamiltonian H of the plant–observer system and its vector L of operators of coupling with W to be computed as

(105) $$H=H+\Gamma +\Phi^{T}JL,\;L=\left[\begin{array}{c} L+\Phi \\ \Psi \end{array}\right].$$

Hence, the internal and output variables $\xi_{1},\ldots ,\xi_{\nu }$ and $\eta_{1},\ldots ,\eta_{m+\mu }$ of the observer in (100) are governed by the QSDEs

(106) $$\begin{array}{cc} d\xi & =G\left(\xi \right)dt-i\left[\xi ,L^{T}\right]dW=\left(i\left[\Gamma ,\xi \right]+\Delta \left(\xi \right)\right)dt-i\left[\xi ,\left[\begin{array}{cc} \Phi^{T} & \Psi^{T} \end{array}\right]\right]d\left[\begin{array}{c} Y\\ \omega \end{array}\right], \end{array}$$

(107) $$\begin{array}{cc} d\eta & =2JLdt+dW. \end{array}$$

Here,

G(ζ):=i[H,ζ]+D(ζ)

is the GKSL generator of the plant–observer system and

$$D\left(\zeta \right)=-\left[\zeta ,L^{T}\right]\Omega L-\frac{1}{2}\left[L^{T}\Omega L,\zeta \right]$$

is the corresponding decoherence superoperator in accordance with (11), (103) and (105). In (106), use is made of the partial decoherence superoperator Δ, which acts on the observer variables as

$$\Delta \left(\xi \right)=-\left[\xi ,\Phi^{T}\right]\Omega \Phi -\left[\xi ,\Psi^{T}\right]℧\Psi -\frac{1}{2}\left[\Phi^{T}\Omega \Phi +\Psi^{T}℧\Psi ,\xi \right]$$

in view of (102)–(105). Now, consider a coherent quantum filtering (CQF) problem formulated as the minimization of the mean square discrepancy

(108) $$Z:=\lim_{t\to +\infty }EZ\left(t\right)⟶\min,\;Z:=\frac{1}{2}{\left(SX-TL\right)}^{T}\left(SX-TL\right)$$

between *r* linear combinations of the plant variables of interest and the entries of the drift part 2JL of the observer output η in (107), as specified by given weighting matrices $S\in R^{r\times n}$ and $T:=\left[\begin{array}{cc} T_{1} & T_{2} \end{array}\right]$ with $T_{1}\in R^{r\times m}$ and $T_{2}\in R^{r\times \mu }$. The criterion process *Z* in (108) is similar to that in (83):

(109) $$Z:=\frac{1}{2}\left[\begin{array}{cc} X^{T} & L^{T} \end{array}\right]\Pi \left[\begin{array}{c} X\\ L \end{array}\right],\;\Pi :=\left[\begin{array}{cc} S^{T}S & -S^{T}T\\ -T^{T}S & T^{T}T \end{array}\right],$$

with *X* being the only subvector of X from (102) which is present in (109). The minimization in (108) is over the observer Hamiltonian Γ and the vector Φ of the observer–plant coupling operators in (101) as functions of the observer variables $\xi_{1},\ldots ,\xi_{\nu }$. This mean square optimal CQF problem extends [20] in that we do not restrict attention to linear observers even if the plant is an OQHO and Ψ depends linearly on the observer variables ξ. Note that the Hamiltonian *H* of the plant and its coupling *L* to the input quantum noise *W* are fixed, as is the coupling Ψ of the observer to the input quantum noise ω; see Figure 1. If $\Gamma_{0}$ and $\Phi_{0}$ are perturbed in the directions

(110) $$K_{0}:=\Gamma_{0}^{\prime },\;M_{0}:=\Phi_{0}^{\prime },$$

consisting of self-adjoint operators representable as functions of the initial observer variables $\xi_{1}\left(0\right),\ldots ,\xi_{\nu }\left(0\right)$, then the corresponding perturbations of the plant–observer Hamiltonian and coupling operators in (105) are

(111) $$H_{0}^{\prime }=K_{0}+M_{0}^{T}JL_{0},\;L_{0}^{\prime }=\left[\begin{array}{c} M_{0}\\ 0 \end{array}\right].$$

By applying Theorem 2 and using (103), (110) and (111), it follows that the corresponding transverse Hamiltonian *Q* for the plant–observer system satisfies the QSDE

(112) dQ=K+MTJL−ImLTΩM0dt+MT0dW=(K−Im((2L+Φ)TΩM))dt+MTdW,

where use is made of the relation Im(LTΩM)=−MTJL following from (1) and the commutativity $[L,M^{T}]=0$. Then, the Gateaux derivative $Z^{\prime }$ of the cost functional in (108) is given by (80), where

jt(Z0′)=Re((TL−SX)TT1M)

in view of (109) and (111) and similarly to (85). In accordance with (82), the expectation of the derivative process ϕ:=i[Q,Z] satisfies the IDE

$${\left(E\phi \right)}^{{}^{\cdot }}=iE\left[Q,G\left(Z\right)\right]+E\chi \left(Z\right),$$

where, in view of (112), the superoperator χ in (69) is given by

χ(Z)=i[K−Im((2L+Φ)TΩM),Z]−2Re([Z,(L+Φ)T]ΩM).

In particular, if both the plant and the unperturbed observer are OQHOs with Hurwitz dynamics matrices (while the perturbations in (110) are not necessarily linear-quadratic), then $Z^{\prime }$ can be found in a form similar to (93) along the lines of Example 3 from Section 6, which is due to the criterion process *Z* in (108) and (109) being a quadratic function of the plant and observer variables in X from (102).

The transverse Hamiltonian method outlined above was used in [41] to show that in the mean square optimal CQF problem for linear quantum plants those observers which are locally optimal in the class of linear quantum observers cannot be improved locally in the sense of the first-order optimality conditions (81) by varying the Hamiltonian and coupling operators of the observer along linear combinations of the Weyl operators associated with the observer variables.

## 8. Concluding Remarks

For a class of open quantum systems governed by Markovian Hudson–Parthasarathy QSDEs, we have introduced a transverse Hamiltonian and derivative processes associated with perturbations of the system Hamiltonian and system–field coupling operators along with the influence of such perturbations on the system dynamics. This provides a fully quantum tool for infinitesimal perturbation analysis of system operators and cost functionals with infinite-horizon formulations that involve averaging over the invariant quantum state of the system. The proposed variational method has been accompanied by detailed proofs and can be used for the development of first-order necessary conditions of optimality in quantum control and filtering problems. We have illustrated these ideas for OQHOs with quadratic performance criteria and the mean square optimal CQF problem. The results of the paper are also applicable to perturbation analysis and variational problems in optimal control and filtering synthesis for more complicated networks of quantum stochastic systems, such as in [7,56].

## Data Availability

Not applicable.

## Abbreviations

The following abbreviations are used in this paper:

ALE	algebraic Lyapunov equation
CCR	canonical commutation relation
CQF	coherent quantum filtering
CQLQG	coherent quantum linear–quadratic Gaussian
GKSL	Gorini–Kossakowski–Sudarshan–Lindblad
IDE	integro-differential equation
ODE	ordinary differential equation
OQHO	open quantum harmonic oscillator
PR	physical realizability
QSDE	quantum stochastic differential equation
